# Short-Range Six-Axis Interferometer Controlled Positioning for Scanning Probe Microscopy

**DOI:** 10.3390/s140100877

**Published:** 2014-01-07

**Authors:** Josef Lazar, Petr Klapetek, Miroslav Valtr, Jan Hrabina, Zdenek Buchta, Onrej Cip, Martin Cizek, Jindrich Oulehla, Mojmir Sery

**Affiliations:** 1 Institute of Scientific Instruments, Academy of Sciences of the Czech Republic, Královopolská 147, Brno 612 64, Czech Republic; E-Mails: shane@isibrno.cz (J.H.); buchta@isibrno.cz (Z.B.); ocip@isibrno.cz (O.C.); cizek@isibrno.cz (M.C.); oulehla@isibrno.cz (J.O.); sery@isibrno.cz (M.S.); 2 Czech Metrology Institute, Brno, Okru žní 31, Brno 638 00, Czech Republic; E-Mails: pklapetek@cmi.cz (P.K.); mvaltr@cmi.cz (M.V.)

**Keywords:** nanometrology, nanopositioning interferometry, AFM, nanoscale

## Abstract

We present a design of a nanometrology measuring setup which is a part of the national standard instrumentation for nanometrology operated by the Czech Metrology Institute (CMI) in Brno, Czech Republic. The system employs a full six-axis interferometric position measurement of the sample holder consisting of six independent interferometers. Here we report on description of alignment issues and accurate adjustment of orthogonality of the measuring axes. Consequently, suppression of cosine errors and reduction of sensitivity to Abbe offset is achieved through full control in all six degrees of freedom. Due to the geometric configuration including a wide basis of the two units measuring in y-direction and the three measuring in z-direction the angle resolution of the whole setup is minimize to tens of nanoradians. Moreover, the servo-control of all six degrees of freedom allows to keep guidance errors below 100 nrad. This small range system is based on a commercial nanopositioning stage driven by piezoelectric transducers with the range (200 × 200 × 10) μm. Thermally compensated miniature interferometric units with fiber-optic light delivery and integrated homodyne detection system were developed especially for this system and serve as sensors for othogonality alignment.

## 1. Introduction

Developments in production technology on the micro-scale, precision manufacturing and especially in the electronic semiconductor industry, have increased the demand for precision measurements of dimensional quantities on a scale below and far below one micrometer. Traditional length metrology relies on optical coherent interferometry where the elementary length to be counted is a wavelength of (visible) light and the light source is a laser with an accurate and stable optical frequency. The lengths that are being measured are large multiples of the wavelength, in an incremental regime offering virtually unlimited dynamic range within the coherence length of the laser source. Measurement far below the wavelength limit together with the need to image and quantify dimensions on the nanoscale generates specific and very complex problems which are the domain of nanometrology, a new discipline of metrology.

Dimensional measurement of objects on the nanoscale needs an imaging method with resolution well below the wavelength of visible light or some sort of a scanning sense with dimensions in the same range as the desired resolution. The concept of instrumentation for nanometrology resulted into a combination of such a microscopy technique with a measuring system capable to deliver the resolution and precision needed on the nanoscale with traceability to the primary length standard. The measurement is done by a coordinate length interferometer linked to the positioning system. In case when objects and structures far below one micrometer have to be resolved there are only a few microscopy techniques available: scanning electron beam microscopy, a dedicated technique called scanning probe microscopy based on the interaction between a probe and the sample, again in a scanning mode, the scanning helium ion microscope, X-ray microscopy, *etc*. Complexity, price and the need for having the object under vacuum conditions disqualifies the electron microscope quite significantly. The standard configuration thus emerges in the form of an atomic force microscope (AFM) with a fixed position probe (tip) and a scanning stage with the sample and interferometers [1,2].

The key features of a primary standard of metrological interferometric position measurement—traceability of the measured quantity—are here secured through the laser source which operates on one of the wavelengths coinciding with transitions in a suitable absorbing media [3]. Stable optical frequency represents a stable wavelength in vacuum; under atmospheric conditions this conversion to wavelength needs an evaluation of the refractive index of air. On the nanoscale where the measuring range may be only tens or hundreds of wavelengths the interpolation of a single fringe becomes a crucial aspect. Precision and stability of the arrangement for measuring within a limited measuring range is often primarily a mechanical problem in which the positioning stage should deliver sensitivity and resolution which is in relation to the measurement. Piezoelectric Transducer (PZT)-driven flexture stages can cover tens or hundreds of micrometers. However, for a larger scale system this becomes a challenge in mechanical engineering [4].

This contribution deals primarily with the aspects of positioning and associated measurement and control of angle (guidance) errors. The presented principle of adjustment of the orthogonality of the measuring beams together with the feedback control of the guidance errors helps not only to reduce the cosine errors, but also contributes to lower sensitivity to the Abbe offset induced errors.

## 2. Concepts

Research effort in the field of nanometrology systems gradually resulted in two concepts distinguished by the measuring range. Scanning over an area below approximately (1 × 1) mm can be done with PZT-driven flexture stages with virtually no backlash, and sensitivity and resolution on the nm level or better [5,6]. The design of the stage relies on flexible joints and suffers moderately from the presence of guidance errors over the scanning range. On a small-range scale they may be considered negligible when the system configuration follows the Abbe principle, the microscope probe coinciding with all the measuring axes [7]. Nanopositioning stages covering ranges over 1 mm or even several cm represent a very complex problem requiring designs with a sophisticated flexture lever system [8–12] or high-quality guidance reducing the backlash [13,14]. Covering a large range with linear positioning actuators needs to overcome the dynamic range either with a stepping principle with incremental steps or magnetic, voice coil actuators operating with displacement integrating the drive current. This can be considered as an equivalent to the incremental principle, extending the dynamic range without sacrificing resolution [15,16]. Even larger range systems have been designed; they can be seen as small 3D coordinate measuring systems targeting the micro-, not the nanoworld [17].

Monitoring/measuring of the stage position in the simplest configuration should include two axes in the horizontal plane; measurement in the vertical axis realizes 3D control [18]. Guidance errors resulting in tilting the stage may introduce cosine errors into the path measured by the interferometers and distortion of the scanned surface on a scale that can be considered not negligible. Full monitoring or control of the stage position requires six-axis measurement where the angular deviations, pitch, roll and yaw are independently measured. Reduction of the guidance (angle) errors can be a part of the mechanical design and the whole system in a full feedback control regime or it may rely on the once measured and guaranteed straightness of motion.

The length measuring system with interferometers is often based on a traditional He-Ne laser source with good primary stability, moderate noise, simplicity and good optical properties of the laser beam. Output power for a single-frequency laser at the 1 mW level is considered enough to feed a limited number of interferometric units, usually not more than three. Stabilization of the optical frequency is derived from a Doppler broadened gain profile of Ne [19]. The choice of an alternative laser source has been presented in [20] where a frequency doubled Nd:YAG laser can be operated in a single-frequency regime with an output power of 100 mW or even more. This can facilitate more complex interferometric setups with additional measuring axes and incorporating fiber-light delivery. Traceability to the primary standard of length has to be secured through linear spectroscopy of molecular iodine in an external absorption cell [21–23] offering sufficient stability.

## 3. Design

### 3.1. Arrangement and Mechanics

**Figure 1.**
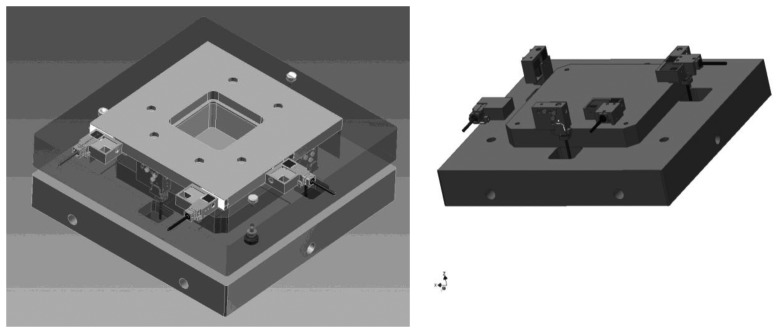
Configuration of the setup (**left**) together with the arrangement of the interferometers in all measuring axes (**right**).

For the project within the framework of the design of the national nanometrology standard we concentrated on a configuration combining an AFM microscope with a commercial nanopositioning stage and a set of interferometers monitoring the position of the sample table in all six degrees of freedom (together with an active feedback control) (Figure 1). The position sensing design uses an arrangement of six independent displacement measuring interferometers. The interferometer assembly consists of three systems measuring vertical z-motion, each of them oriented 120° with respect to each other and the center of the stage. They measure vertical displacement and pitch and roll angles are evaluated from their differences. Interferometers monitor a single x displacement and y-position is measured by the two which gives the information about the third angle rotation according to the vertical axis (the yaw angle) [24]. The nanopositioning x-y-z stage is a PZT-driven system with 200 μm travel range in both x and y axes and 10 μm range in the vertical z axis. The stage is equipped with capacitive sensors with nm resolution and is designed to operate in a closed loop displacement control eliminating the effects of PZT nonlinearity and hysteresis. This can be considered as a small range system where we concentrated more on the resolution and sensitivity for measuring small samples.

**Figure 2.**
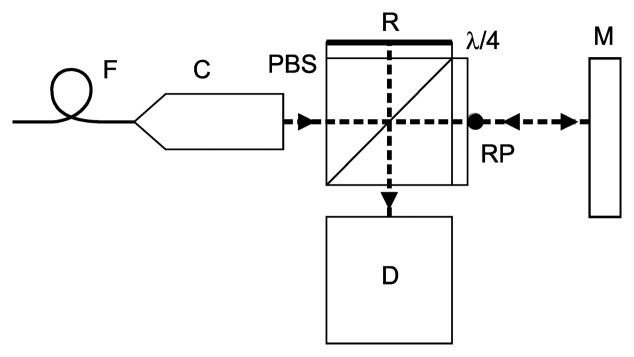
Schematics of the single-pass interferometer with a plane mirror reflector. PBS: polarizing beam splitter; M: plane mirror; C: collimator; λ/4: retardation plate; D: detection unit; F: optical fiber; R: reflective surface; RP: reference point.

The design of the mechanics is derived from previous testing arrangements [25–27]. The configuration consists of two frames, one representing a base-plate holding the nanopositioning stage and the second being the metrology frame enclosing the positioning stage, the mount for the set of all six interferometers. The metrology frame is made from a material with low thermal expansion. We opted for Invar alloy, because of reduced cost and easier manufacturing. The interferometric units are mounted into frames made by milling, again from Invar alloy, the same material as that of the frame. Attachment of the unit is fixed at the reference point (RP, see Figure 2) and allows two angle adjustments through micropositioning screws. The sample holder plate is also made from Invar to eliminate thermal expansion effects—the thermal drift of the frame is compensated by the plate.

## 3.2. Interferometers

The set of interferometers was designed and manufactured especially for this purpose. The optical setup was chosen as a simple plane-mirror interferometer. Our motivation was driven primarily by reduction of the thermal effects where the reference and measuring beam paths in the bulk optics were kept the same and thus compensating themselves. Otherwise, thermal expansion of the glass components introduces additional uncertainty. The trade-off due to reduced resolution of the single-pass configuration was considered negligible. The configuration of each of the interferometric units is shown in Figure 2. Referencing of the single-pass interferometers is to a point (RP) which is the location where the beam first leaves the glass of the optics. Thermal expansion of the optics is thus effectively compensated by adequate increase of the reference beam path. The point RP represents a point of attaching the unit to the frame.

**Figure 3.**
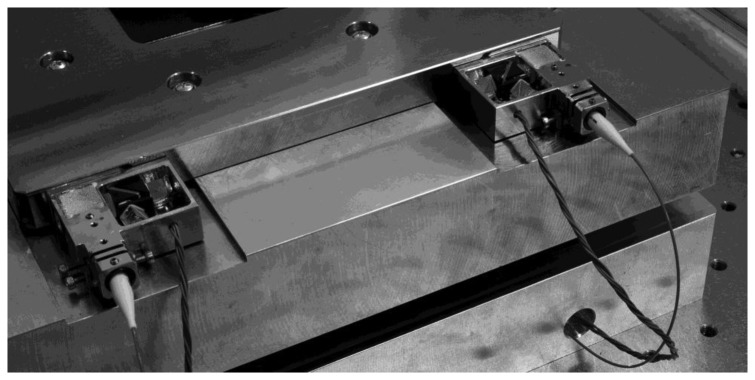
Photo of a pair of the interferometer units with homodyne detection, adjustment screws and fiber light delivery.

The second motivation for a simple plane-mirror interferometer is its sensitivity to tilt of the measuring mirror. The sensitivity of the output contrast of the interference signal is enough to resolve tilt of a few arc seconds. This effect was exploited for adjustment of orthogonality of the measuring axes [28]. This is crucial for elimination of cosine errors. Plane-mirror multipath interferometers with corner-cube reflectors in both measuring and reference paths are able to compensate for angle deviation of the target mirror with no loss of contrast of the interference fringes. The plate—sample holder which is equipped with six target mirrors was tested for parallelism and orthogonality with the help of autocollimator. The measurement has proven that the mirror angle misalignment is on the level of 10 arc seconds. Due to the adjustment of the interferometers through a visible drop in fringe contrast we are able to ensure the orthogonality of all measuring axes at the level of a few tens of arc seconds. Figure 3 shows one interferometeric unit of our miniaturized design in an Invar frame together with homodyne detection.

The laser light delivery to the interferometers is fully fiber optic via a single-mode polarization maintaining fiber and a polarization maintaining splitter with one input and eight outputs where the light delivery of the iodine cell was via a fiber as well. The 8th output was reserved for a 7th interferometer which is intended to monitor the vertical positioning of the AFM tip. Resolution of the interferometric detection and data processing system here is 10 bit with 1 Least Significant Bit (LSB) being the 1/1,024th of one cycle of the interferometric signal. The λ/2 resolution of the interferometric system itself results in a final value of λ/2,048 or a resolution of 260 pm for the wavelength of 532 nm employed. The small horizontal and even smaller vertical positioning range raises the importance of the linearity of the scale. Linearity of the fringe division is further improved by a software linearization algorithm included directly into the signal processing hardware of the interferometer signal [24,25].

## 4. Orthogonality and Angle Control

Plane-mirror multipath interferometers with corner-cube reflectors in both measuring and reference paths are able to compensate for angle deviation of the target mirror with no loss of contrast of the interference fringes. Our simple design of the interferometric units with plane-mirror configuration results in high angular sensitivity of the interference signal to the mirror tilt. This produces angle deviation of the wavefronts of the reference and measuring beams and reduces significantly the contrast of the interference signal detected by large-surface photodetectors over several mm^2^ surfaces. The sensitivity is enough to resolve tilt of a few arc seconds. This effect was exploited for adjustment of orthogonality of the measuring axes.

This approach gave us the chance to adjust the orthogonality of multiaxis measuring system with resolution at the few arc second level. Otherwise it would have to be adjusted by other, less accurate means, e.g., by monitoring of the beam position over a larger distance. e.g., the 10 arc s precision would need a spatial resolution of 50 μm over 1 m distance. This approach relies, of course, on orthogonality of the reflective surfaces of the sample holder. In our case it is equipped with six target mirrors. They were tested for parallelism and orthogonality with the help of autocollimator. The measurement has proven that the mirror angle misalignment is on the level of 10 arc s.

**Figure 4.**
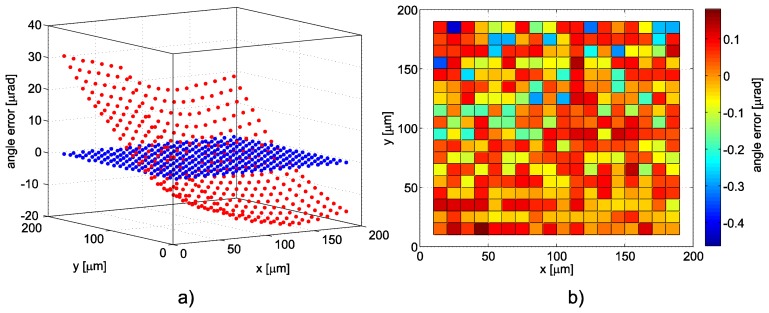
Angle guidance performance of the free-running stage. (**a**) without feedback control of angle—red; with full feedback control of the motion of the stage guidance errors—blue; (**b**) angle error over the whole field of motion with feedback control in detail.

We proposed a system with full control of all six degrees of freedom where the stage covers the positioning range with guidance errors up to approximately 10 μrad. We added a set of PZT actuators combining vertical and horizontal sheer motion for control of all the tilts with a range large enough to cover this scale. The positioning performance of the free-running stage (without feedback control of angle via additional PZTs) was tested through evaluation of guidance errors. The maximum deviation of the yaw angle (along the vertical axis) proved to be below 10 μrad and the pitch and roll max. at approx. 5 μrad along the travel range. With full feedback control of the motion of the stage the guidance errors were kept below the 100 nrad level (Figure 4).

The measurement of the position is performed in all six degrees of freedom and evaluation of the angle deviations through independent parallel measuring beams. Presence of a cosine error caused by misalignment of orthogonality (parallelism) in one measuring axis contributes not only directly to the error of position sensing, but also results in multiplicative guidance angle errors. This can be considered really negligible and with orthogonality at the few arc second level it can be below fm level. The importance of orthogonality would rise with the travel range.

The reduction of the influence of the Abbe offset induced errors may be achieved through reduction of the guidance angle errors or through minimizing the Abbe offset. In case of a combination of a scanning probe microscope and an interferometer controlled positioning this means unifying the intersection of the measuring axes (beams) with the tip of the probe. This may rely on the precision of manufacturing of the mechanics or may be done by additional alignment. Still the precision below 0.1 mm is not simple to achieve. To further reduce the guidance errors through even more accurate control of the stage positioning is worth the effort. An overview of the uncertainty budget contributions related to the orhogonality, guidance and Abbe offset induced errors is presented in Table 1. The uncertainty budget considering all other parameters has been presented in [25]. Values for the compensated and not compensated guidance errors are derived from the measurement in Figure 4. Uncertainty estimates are expressed only for the displacement measuring and positioning system, and the Abbe offset induced errors are considered for the offset a = 0.1 mm. Uncertainty estimates are calculated for the 200 μm measuring range.

## 5. Conclusions

Design and performance of the system presented here are a part of the effort to establish a national standard for nanometrology. This is to be operated by the Czech Metrology Institute (CMI) in Brno, Czech Republic. The instrument was designed as a small range scanning stage with full six-axis interferometric measurement of the position of the sample holder employing six independent interferometers. Due to the geometric configuration with a wide basis of the two units measuring in the y-direction and the three measuring in the z-direction the angular resolution of the whole setup goes down to tens of nanoradians [29]. The positioning range is limited by a commercial stage driven by piezoelectric transducers with an overall of range 200 × 200 × 10 μm.

The novel approach of our design is represented by especially designed interferometer units with homodyne detection and thermal expansion compensation. The units with flat mirror configuration perform very high sensitivity to the tilt of the target mirror. Together with accurate alignment of the mirrors of the sample holder this enables the alignment of orthogonality of the measuring beams with precision on the level of few tens of arc seconds by angle adjustment of the measuring axis of each unit to achieve maximum contrast of the interference signal.

We demonstrated that employing the full six-axis control of the positioning stage in a servo-loop following the interferometers outputs the guidance errors could be efficiently compensated. This allows us not to pursue the Abbe principle with such a high precision. Distortion of the scanned and measured image of the sample is thus eliminated.

## Figures and Tables

**Table 1.** Uncertainty estimates for the positioning and displacement measuring system (horizontal).

**Quantity**	**Standard** **Uncertainty**	**Sensitivity Coefficient**	**Uncertainty** **Contribution**
Orthogonality errors	5″	2·x·(1 − cos(α))	0.1 pm
Guidance angle errors (no compensation)	10 μrad ≈ 2″	x·(1 − cos(α))	0.01 pm
Guidance angle errors (with compensation)	1 μrad ≈ 0.2″	x·(1 − cos(α))	0.1 fm
Abbe offset induced errors (no compensation = α < 10 μrad)	0.1 mm	a·sin(α)	1 nm
Abbe offset induced errors (with compensation α < 1 μrad)	0.1 mm	a·sin(α)	0.1 nm
